# An Evaluation of the Policy Context on Psychosocial Risks and Mental Health in the Workplace in the European Union: Achievements, Challenges, and the Future

**DOI:** 10.1155/2015/213089

**Published:** 2015-10-18

**Authors:** Stavroula Leka, Aditya Jain, Sergio Iavicoli, Cristina Di Tecco

**Affiliations:** ^1^Centre for Organizational Health & Development, School of Medicine, University of Nottingham, Jubilee Campus, Wollaton Road, Nottingham NG8 1BB, UK; ^2^Nottingham University Business School, University of Nottingham, Jubilee Campus, Wollaton Road, Nottingham NG8 1BB, UK; ^3^Italian Workers' Compensation Authority (INAIL), Department of Occupational and Environmental Medicine, Epidemiology and Hygiene, Via Fontana Candida 1, Monteporzio Catone, 00040 Rome, Italy

## Abstract

Despite the developments both in hard and soft law policies in the European Union in relation to mental health and psychosocial risks in the workplace, a review of these policies at EU level has not been conducted to identify strengths, weaknesses, and gaps to be addressed in the future. Keeping in mind that the aim should be to engage employers in good practice, ideally such policies should include key definitions and elements of the psychosocial risk management process, covering risk factors, mental health outcomes, risk assessment and preventive actions, or interventions. The current paper aims to fill this gap by reviewing hard and soft law policies on mental health in the workplace and psychosocial risks applicable at EU level and conducting a gap analysis according to a set of dimensions identified in models of good practice in this area. Our review of ninety-four policies in total revealed several gaps, especially in relation to binding in comparison to nonbinding policies. These are discussed in light of the context of policy-making in the EU, and recommendations are offered for future actions in this area.

## 1. Introduction

It is generally accepted that “work is good for you,” contributing to personal fulfillment and financial and social prosperity [1]. There are economic, social, and moral arguments that, for those who are able to work, “work is the best form of welfare” [2–4] and is the most effective way to improve the well-being of these individuals, their families, and their communities. Moreover, for people who have experienced poor mental health, maintaining, or returning to, employment can also be a vital element in the recovery process, helping to build self-esteem, confidence, and social inclusion [5]. A better working environment can help improve employment rates of people who develop mental health problems. Not doing this puts additional costs on governments that have to provide social welfare support for people who would prefer to be in employment.

There is also growing awareness that (long-term) unemployment is harmful to physical and mental health, so it could be assumed that the opposite must be true that work is beneficial for health. However, that does not necessarily follow [1]. Work is generally good for your health and well-being, provided you have “a good job” [1, 6]. Good jobs are obviously better than bad jobs, but bad jobs might be either less beneficial or even harmful. In fact, a study by Westerlund et al. [7] shows an improvement in fatigue and depressive symptoms associated with the retirement event, especially for those exposed to the worst work environment.

This paper focuses on mental health in the workplace and adopts a comprehensive approach and an inclusive definition of mental health with a focus not only on (the absence of) mental health disorders but also on positive state of psychological well-being. This approach underlines the need to address mental health in its totality by recognising interrelationships among risks to mental health, subthreshold conditions of poor psychological health and well-being (such as stress), which may have not yet resulted in a diagnosed mental health disorder but may severely affect their expression, and diagnosed mental health disorders. According to this perspective, efforts to tackle mental ill health should not focus on particular problems in isolation, such as depression, for example, but they should seek to put in place policies and practices that will tackle a wider range of risk factors to mental health by appropriate interventions. These should prioritise prevention and tackling problems at source while also developing awareness and facilitating treatment.

This paper focuses on the workplace where one of the key states of suboptimal mental health that can have severe consequences is work-related stress. Work-related stress is the response people may have when presented with work demands and pressures that are not matched to their knowledge and abilities and which challenge their ability to cope [8]. The European Commission [9] defined stress as a pattern of emotional, cognitive, behavioural, and physiological reactions to adverse and noxious aspects of work content, work organisation, and work environment. In the framework agreement on work-related stress [10], stress is defined as a state, which is accompanied by physical, psychological, or social complaints or dysfunctions and which results from individuals feeling unable to bridge a gap with the requirements or expectations placed on them.

A substantial body of evidence is now available on work-related risks that can negatively affect both mental and physical health with an associated negative effect on business performance and society [11]. Although risks in the physical work environment can have a direct negative effect on mental health that is accentuated by their interaction with risks in the psychosocial work environment. In addition, psychosocial hazards (also often termed work organisation characteristics or organisational stressors) have been shown to pose significant risk and have a negative impact on mental health, mainly through the experience of work-related stress [11, 12]. These hazards are closely associated with the changing nature of work.

### 1.1. The Prevalence and Impact of Work-Related Psychosocial Risks and Mental Ill Health in the EU

In 2005 and again in 2010, every fourth participant of the European Working Conditions survey believed that their health is at risk due to work-related stress [13]. Even from early 2000, studies suggested that between 50 and 60% of all lost working days have some link with work-related stress [14] leading to significant financial costs to companies as well as society in terms of both human distress and impaired economic performance. In 2002, the European Commission reported that the yearly cost of work-related stress and related mental health problems in 15 Member States of the pre-2004 EU was estimated to be on average between 3 and 4% of the gross national product, amounting to €265 billion annually [15].

In addition, the estimates for the proportion of the workforce in Europe that may be living with a mental health problem at any one time range from one in five [16] to two in five [17], with a lifetime risk of at least two in five [16]. In the EU-27, it was found that 15% of citizens had sought help for a psychological or emotional problem, with 72% having taken antidepressants [18].

A report by EU-OSHA summarized the economic costs of work-related stress illnesses. It reported that, in France, between 220,500 and 335,000 (1–1.4%) people were affected by a stress-related illness which cost the society between €830 and €1.656 million; in Germany, the cost of psychological disorders was estimated to be EUR 3,000 million [19]. Each case of stress-related ill health has been reported to lead to an average of 30.9 working days lost [20]. Estimates from the UK Labour Force Survey indicate that self-reported work-related stress, depression, or anxiety accounted for an estimated 11.4 million lost working days in Britain in 2008/09 [21]. This was an increase from earlier estimates, which indicated that stress-related diseases are responsible for the loss of 6.5 million working days each year in the UK, costing employers around €571 million and society as a whole as much as €5.7 billion. A recent study concluded that the “social cost” of just one aspect of work-related stress (job strain) in France amounts to at least 2-3 billion euros, taking into account healthcare expenditure related to absenteeism, people giving up work, and premature deaths [22].

### 1.2. Policies on Psychosocial Risks and Mental Health in the Workplace

Psychosocial risks and their management are among employers' responsibilities as stipulated in the Framework Directive 89/391/EEC on Safety and Health of Workers at Work as it obliges employers to address and manage all types of risk in a preventive manner and to establish health and safety procedures and systems to do so. In addition to the Framework Directive, a number of policies and guidance of relevance to mental health have been developed and are applicable to the European level. These include both legally binding instruments (such as EU regulations, Directives, decisions, and national pieces of legislation) and other “hard” policies (such as ILO conventions) developed by recognised national, European, and international organisations as well as nonbinding/voluntary policies (or “soft” policies) which may take the form of recommendations, resolutions, opinions, proposals, conclusions of EU institutions (Commission, Council, and Parliament), the Committee of the Regions, and the European Economic and Social Committee, as well as social partner agreements and frameworks of actions, and specifications, guidance, campaigns, and so forth initiated by recognised European and international committees, agencies, and organisations.

Regulatory instruments of relevance to mental health and psychosocial risks are applicable to all EU member states. However, even though each of these regulations addresses certain aspects of mental health and/or the psychosocial work environment, it should be noted that the terms “mental health,” “stress,” and “psychosocial risks” are not mentioned explicitly in most pieces of legislation [23]. The main example in this respect is the Framework Directive 89/391/EEC on Safety and Health of Workers at Work. Even though the Directive asks employers to ensure workers' health and safety in every aspect related to work, “addressing all types of risk at source,” it does not include the terms “psychosocial risk” or “work-related stress.” However, it does require employers to “adapt the work to the individual, especially as regards the design of workplaces, the choice of work equipment, and the choice of working and production methods, with a view, in particular, to alleviating monotonous work and work at a predetermined work rate, developing a coherent overall prevention policy which covers technology, organization of work, working conditions, social relationships, and the influence of factors related to the working environment.”

The Directive further specifies that “health surveillance should be provided for workers according to national systems. Particularly sensitive risk groups must be protected against the dangers which specifically affect them.” In this sense, there is an indirect reference to, and provision for, risks related to mental health at work. This is also the case for the Directive on organisation of working time (93/104/EC), while the Council Directive on work with display screen equipment (90/270/EEC) actually refers to “problems of mental stress” in the context of risk assessment. It should be noted here that, in some EU member states, the national regulatory frameworks are more specific than the key EU occupational health and safety Directives and do make reference to psychosocial risks and work-related stress.

A debate has been taking place in the scientific and policy literatures about the lack of clarity in regulatory frameworks and related guidance on mental health at work and the management of psychosocial risks (e.g., [24–26]). A recent European Survey of Enterprises on New & Emerging Risks (ESENER) which covered over 28,000 enterprises in 31 countries across Europe has revealed that even though work-related stress was reported among the key OSH concerns for European enterprises, only about half of the establishments surveyed reported that they inform their employees about psychosocial risks and their effects on health and safety and less than a third had procedures in place to deal with work-related stress. The findings of the survey also showed that 42% of management representatives consider it more difficult to tackle psychosocial risks, compared with other safety and health issues. The most important factors that make psychosocial risks particularly difficult to deal with were reported to be “the sensitivity of the issue,” “lack of awareness,” “lack of resources,” and “lack of training” [27]. The second edition of EU-OSHA's ESENER collected similar information on OSH management and workplace risks, with a particular focus on psychosocial risks, from almost 50,000 enterprises in 36 countries across Europe. Recently published, first findings have revealed that psychosocial risk factors are reported as more challenging than other risks. The most important factors that make psychosocial risks particularly difficult to deal are “lack of information” and “lack of adequate tools to deal with the risk effectively” as perceived by almost one in five establishments reporting “dealing with difficult customers” or “experiencing time pressure” [28].

Similar findings have also been found in stakeholder surveys, which report that many stakeholders still perceive workplace hazards as primarily relating to physical aspects of the work environment. Furthermore, where issues relating to mental health are reported to be important OSH concerns, there are significant differences among the perception of stakeholders in different countries in the EU [29, 30]. These differences in perception (in terms of perspectives, priorities, and interests) of mental health at work between social actors, particularly between employers' organisations and trade unions, are a challenge for effective social dialogue on these issues and for the effective implementation of recently introduced voluntary policy initiatives for the management of psychosocial risks such as the European framework agreements on work-related stress and on harassment and violence at work [31].

In addition to the regulatory instruments, a significantly larger number of “soft” policy initiatives of relevance to mental health and psychosocial risks in the workplace have been developed and implemented at the EU level. An EU-OSHA report on workplace mental health promotion cites some of the recent policy documents and initiatives within the EU relevant to mental health at work [32]:

1. Lisbon Strategy: EU goal for economic growth and competitiveness;
2. Community Strategy on Health and Safety at Work, 2007–2012;
3. Commission White Paper “Together for Health”;
4. Framework Agreement on Work-related Stress;
5. Framework Agreement on Harassment and Violence at Work;
6. The Mental Health Pact.

The EU-OSHA report highlights the wide scope of policies in this area, which range from broad EU strategies and public health policies to social dialogue initiatives. In addition to these, other policy initiatives of relevance to mental health and psychosocial risks in the workplace include the setting-up of formalised stakeholder committees, EU level campaigns, policies on managing disability, and initiatives by organisations such as the WHO and ILO. Many of these soft law initiatives and policies are directly relevant to mental health in the workplace, psychosocial risks, work-related stress, and their management. However, very little evaluation has been conducted on hard and soft law policies in Europe.

An evaluation of the implementation of the Framework Directive conducted a decade ago indicated that the tasks of risk assessment, documentation, and supervision are not universally spread, even in member states with a tradition based on prevention [33]. The report also highlighted that, where procedures were in place in organisations, they generally focused on obvious risks where long-term effects (e.g., mental health) as well as risks that are not easily observed were being neglected. There was also hardly any consideration of psychosocial risk factors, and risk assessments were often being considered to be a one-time obligation lacking continuity where the efficiency of the measures was not sufficiently monitored by employers. The findings of the evaluation indicated that much still needed to be done as regards psychosocial risks such as work control and work organisation, preventing unreasonably intense work pace, and repetitive work. This suggested an insufficient application of some of the general principles of the prevention foreseen in the Framework Directive 89/391 [25].

Concerning the evaluation of the framework agreements for work-related stress and for harassment and violence at work, the main activities that followed the signing of the agreements were their translation in national languages [34, 35]; however, they did act as catalysts for the implementation of new or updated legislation in some countries (e.g., the Czech republic and Italy). It should be noted that there is a rather mixed picture regarding the state of European social dialogue in the area of psychosocial risks at work and, as a result, serious questions have been raised in the literature as to the appropriateness and effectiveness of “autonomous, or voluntary, agreements” [36]. Indeed, Ertel and colleagues [31] call for focused activities at European level to harmonize stakeholder perspectives on the issue of psychosocial risk factors and work-related stress.

As discussed before, in some EU member states (e.g., Sweden, Belgium, Italy, Germany, the Czech Republic), legislation is even more specific than EU law and makes direct reference to work-related stress, bullying and harassment, or psychosocial risks [6], although in very few countries stress-related diseases are included in official lists of occupational diseases. In addition, good practice examples in this area exist in a number of member states. Some examples include the Management Standards in the UK and Italy, Work Positive in Ireland, the Work and Health Covenants and Catalogues in the Netherlands, ISTAS in Spain, SOBANE in Belgium, the tools developed by INRS and ANACT in France, and EU-OSHA's online simple risk assessment tool for SMEs, OiRA [37]. Indeed, Iavicoli et al. [38] have called for a critical evaluation of efforts employed so far to address psychosocial risks and mental health in the workplace to be conducted in order to develop an approach at European level that will allow both flexibility at national level and a certain level of benchmarking across members states in terms of relevant data and good practices applied.

### 1.3. The Current Study

Since policies are an important starting point in addressing key issues, it is first important to identify the key elements policies in this area should address. Keeping in mind that the aim should be to engage employers in good practice, ideally such policies should include elements of the psychosocial risk management process, covering risk factors, mental health outcomes, risk assessment and preventive actions, or interventions. However, a review of hard and soft law policies at EU level along these dimensions has not been conducted to identify strengths and weaknesses and gaps to be addressed in the future. The current paper aims to fill this gap by reviewing hard and soft law policies on mental health in the workplace and psychosocial risks applicable at EU level and conducting a gap analysis according to a set of dimensions identified in models of good practice in this area. In particular, the review and gap analysis has used the PRIMA-EF model as a guide [11] which highlights the key steps and principles of the psychosocial risk management process.

## 2. Method

The first step in the process was to identify all relevant hard and soft law policies of relevance to mental health in the workplace and psychosocial risks. This was based on reviews previously conducted by the members of the research team (see [23]). This review was updated to include sectoral Directives as well as policies of relevance to mental health in the workplace more broadly speaking (and not solely psychosocial risks and work-related stress). The review therefore included not only general and specific health and safety policies but also policies relating to working hours, part-time work, temporary work, parental leave, discrimination, organizational restructuring (job insecurity), and so forth.

On the basis of a set of defined criteria in the form of a policy scorecard (see Table 1), a gap analysis was carried out to examine the extent to which the current EU policy framework covered issues relating to mental health in the workplace. Each policy (regulatory or nonbinding) was scored on a scale of “0–5” on the basis of its relevance/applicability to and/or coverage of dimensions relating to mental health at work. The five dimensions were chosen on the basis of good practice guidance [11] and according to the comprehensive definition on mental health in the workplace adopted in this study. The five dimensions were reference to mental health to in the objectives and scope of the policy, coverage of exposure factors, mental health problems/disorders at work and related outcomes, risk assessment aspects, and preventive actions in relation to mental health in the workplace. Policies which did not cover or refer to mental health at work were given a score of 0 while policies which were directly relevant and comprehensively covered each dimension were given a score of 5.

Each policy was reviewed by four researchers working in pairs to analyze the policy content and “assign scores” on the established criteria. To ensure interrater reliability, a method for qualitative data analysis for applied policy research proposed by Ritchie and Spencer [39] was used where both pairs of researchers reviewed the policy text for hard and soft law policies independently. The assigned scores were then discussed and reflected upon by all four researchers. Where disagreement arose, an independent expert reviewed the policy in question. The final assigned score on each dimension was established by consensus in terms of the majority.

## 3. Results 

Table 2 presents the policy scorecard of regulatory instruments of relevance to mental health and psychosocial risks applicable to the EU member states. These include European Union Directives and ILO conventions. These regulations address certain aspects of mental health and/or the psychosocial work environment; however, most policies scored 5 or below across the five dimensions highlighting a lack of coverage and specificity. Directive 89/391/EEC, the European Framework Directive on Safety and Health of Workers at Work, received the highest score [13] along with a Directive 2010/32/EU, implementing the Framework Agreement on prevention from sharp injuries in the hospital and healthcare sector concluded by HOSPEEM and EPSU. Directive 2010/32/EU is, however, applicable only to the healthcare sector.

Table 3 presents the policy scorecard of voluntary policy initiatives, which directly address mental health and psychosocial risks in the workplace. These policy initiatives were scored much more favourably as compared to binding/regulatory policies. Eleven policy initiatives had overall scores of 20 or more, indicating that many policy initiatives explicitly referred to mental health and psychosocial risks in the workplace in the objectives and scope of the policy and sufficiently or comprehensively covered aspects relating to exposure factors, mental health problems at work and related outcomes, aspects of risk assessment and preventive actions.

**Figure 1:**
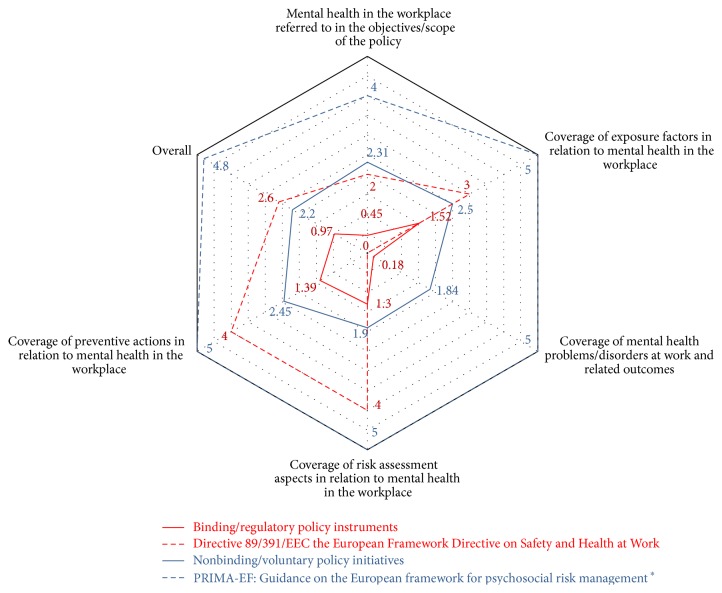
Gap analysis on coverage dimensions across binding and nonbinding policies. ^*∗*^Note: The EC 1999 Guidance on work-related stress: Spice of life or kiss of death? EU-OSHA 2002 Guidance on How to Tackle Psychosocial Issues and Reduce Work-Related Stress; ILO 1986 Guidance on Psychosocial Factors at Work: Recognition and Control; and the ILO, 2012, SOLVE Guidance had the same score as the PRIMA-EF guidance on each dimension.

Further analysis explored the average coverage of each of the review dimensions across binding and nonbinding policies (see Figure 1). The solid lines in Figure 1 depict average scores of all binding/regulatory policies and all nonbinding/voluntary policy initiatives, while the dotted lines plot the scores of the highest scored binding policy (Directive 89/391/EEC) and nonbinding policy (PRIMA-EF guidance) on each dimension. It is clear that nonbinding/voluntary policy initiatives are more explicit in their reference to mental health and psychosocial risks in the workplace in the objectives and scope of the policy and cover aspects relating to exposure factors, mental health problems at work and related outcomes, aspects of risk assessment and preventive actions, in more detail as compared to binding/regulatory policies overall and in each of the five dimensions. A comparison of the highest scored binding and nonbinding policies also indicate the same finding.

## 4. Discussion

From the review and gap analysis presented on regulatory and voluntary policy initiatives, it is possible to make some observations. Keeping in mind that the policies reviewed are those that apply at European Union level alone (and not member state policies), it is encouraging to see that a large number of relevant policies exist both of a binding and a voluntary nature. Our review covered thirty-four regulatory and sixty voluntary policy initiatives and, in the case of the latter, the number is likely to steadily increase year on year, since mental health and psychosocial risks in the workplace represent a constant priority in Europe and other countries. The review and gap analysis also shows that higher scores have been assigned to nonbinding (or soft law) policies. Indeed no binding policy achieved a score higher than 2.5, while several voluntary policies achieved scores of 4.5 and higher. This certainly reflects the focus of the specific policies as well as their development process and regulatory nature.

Binding policies are the outcome of lengthy negotiations among various stakeholders. Depending on the issue at hand and the extent to which it is considered controversial, the text of the policy will reflect this. It is not surprising to see less coverage of the review dimensions in binding regulation due to the lack of agreement on psychosocial issues among social partners and their perceived “sensitivity,” however gaps in terms of definitions and terminology cannot be ignored. As discussed previously, these issues have been raised in the literature and there are several calls for clarifying the text of binding policies further through the inclusion of specific terms (such as work-related stress, psychosocial risks, and mental health at work). While from our review it can be seen that there is more coverage of exposure factors, risk assessment, aspects and preventive action, this is still limited in comparison to nonbinding policies.

On the other hand, voluntary policies are often developed by experts alone and usually do not involve negotiation but rather a review process (which could still involve all relevant stakeholders). They are more focused in terms of addressing specific issues and often aim at providing guidance on implementing good practice. As a result, terminology in these policies is more specific and inclusive and coverage of key elements is more extensive (as also shown in Figure 1).

It is important to note that this review provides an overview on the basis of the content of policies in this area. However, it does not draw any conclusions on the uptake and impact of these policies in practice. Two key issues concern the extent to which these policies offer specific guidance on managing risks in relation to mental health in the workplace to enable organisations (and especially small and medium-sized enterprises) to implement a preventive framework of action and whether existing policies have actually fulfilled expectations in practice in the area of mental health in the workplace. Naturally, one would expect that the binding nature of regulation means that they would be adopted more in practice. However, recent findings suggest that although occupational health and safety legislation is seen by European employers as a key driver to address health and safety issues, it has been less effective for the management of psychosocial risks and the promotion of mental health in the workplace [27, 40].

In relation to voluntary policy instruments, there is the question of whether they have been effective in supporting the implementation of existing legislation and in guaranteeing quality with regard to the “essential requirements” established by European binding policies. Unfortunately, very little evaluation exists in this area and it is difficult to draw any meaningful conclusions. A meaningful example in this direction comes from the last report on the implementation of the European framework agreement on work-related stress signed by the representatives of European social partners [41]. This agreement has had important positive effects, accelerating social dialogue and the development of policies on work-related stress in most of the EU countries. After 10 years from signing, it has been implemented in most of the countries of the EU in different ways: being translated in 8 countries, leading to the signing of national agreements with social partners in 9 countries, being implemented in national legislation in 9 countries. In addition, an evaluation of soft law would not be sufficient unless national policies and relevant initiatives were also taken into account. Traditions of national level research into occupational health and safety in general and specifically in relation to psychosocial risks and their management, national discourses on health and safety definitions and priorities socially and politically, and the practical application of research knowledge to workplace practice are also important determinants of action in this area [42]. However, ESENER results do indicate low action of European organisations and further need for guidance and support [43]. Given the number of voluntary initiatives, one would expect further uptake at company level, and questions in relation to effectively communicating these to organisations/employers in a user-friendly manner, or highlighting positive benefits, are relevant in order to engage them in action.

According to the findings of our review and the wider context of policy-making in Europe, if the status quo concerning the policy context to mental health in the workplace is maintained, it is likely that a number of initiatives will continue to take place across the EU in this area, given the impact of mental ill health on individuals, organisations, and society. However, there is uncertainty as to whether they will achieve the desired outcomes. Although there have been a number of policy initiatives for more than ten years in the EU, awareness in relation to mental health in the workplace and the importance of preventive action still seems to be lacking on the whole and especially among SMEs. This is despite the available data that map the prevalence and impact both of risk factors and mental ill health outcomes. In addition, despite the fact that the Framework Directive 89/391/EEC covers all types of risk to workers' health and as the framework agreement on work-related stress clarifies, including work-related stress, there still appears to be limited awareness of this provision both by employers and other key stakeholders such as policy makers and inspectors in different countries. Limited awareness and expertise on how to conduct inspections on psychosocial risks associated with mental ill health were among the key drivers for the 2012 SLIC campaign [44]. However, with widespread budget cuts in the public sector, inspections in many countries are becoming more reactive in nature [37].

In light of this, it would be advisable to revisit the content of the Framework Directive in relation to psychosocial risks and mental health in the workplace to provide further clarity and harmonize terminology across other key pieces of legislation accordingly. In absence of this, a clear interpretation of the legal provisions in this area by the European Commission would be needed. There is also more scope for better and closer collaboration and coordination to achieve maximum impact in a cost-effective manner at EU institutional level since several policy initiatives and studies have been implemented in this area, for example, from different EC Directorate Generals, the European Parliament, and the European Agency for Safety & Health at Work. Finally, it is important that employer responsibility is strengthened and awareness is further developed both in relation to the policy framework on mental health in the workplace and specific preventive measures that should be introduced to promote mental health, and the promotion of soft law initiatives is essential towards this end.

## 5. Conclusions

Mental health and psychosocial risks in the workplace have been recognised as priorities in occupational health and safety in the European Union for at least two decades. A number of hard and soft law policies of relevance to them have been developed over the years that have promoted awareness and action among policy makers, social partners, organisations, and indeed individual workers. This paper aimed to provide a review and gap analysis of hard and soft law policies applicable at EU level in this area and offer recommendations for the future. Our review of ninety-four policies across five key dimensions revealed several gaps, especially in relation to binding in comparison to voluntary policies. According to the findings of our review and the wider context of policy-making in Europe, if the status quo as concerns the policy context to mental health in the workplace is maintained, it is uncertain whether desired outcomes will be achieved in practice since awareness in relation to mental health in the workplace and the importance of preventive action still seems to be lacking. It is therefore recommended that key EU legislation is made clearer in this area by either including specific terminology and harmonizing it across other key pieces of legislation accordingly or by the development of a clear interpretation of the legal provisions in this area by the European Commission. It is also recommended that there is a better coordination at EU institutional level to achieve maximum impact and not isolated and indeed competitive and non-cost-effective efforts. Finally, it is important that soft law initiatives continue to be promoted to strengthen employer awareness, responsibility, and engagement in preventive actions.

## Figures and Tables

**Table 1:** Policy scorecard: key dimensions and scoring criteria.

Key dimensions	0	1	2	3	4	5
Mental health in the workplace referred to in the objectives and scope of the policy	Not covered by the general objectives or scope of the policy	Covered in principle but not effectively addressed	Only implicitly covered by the objectives/scope of the policy	Partially covered by the objectives/scope of the policy	Sufficient coverage but lack of definitions of key terms within the policy	Comprehensively covered by the general objective or scope of the policy

Coverage of exposure factors in relation to mental health in the workplace	No reference to or acknowledgement/coverage of exposure factors in relation to mental health in the workplace	Covered in principle but not effectively addressed	Only implicit acknowledgement/coverage of some exposure factors in relation to mental health in the workplace	Partial acknowledgement/coverage of exposure factors in relation to mental health in the workplace	Sufficient coverage but lack of specificity on exposure factors in relation to mental health in the workplace	Comprehensive coverage of exposure factors in relation to mental health in the workplace

Coverage of mental health problems/disorders at work and related outcomes	No reference or acknowledgement/coverage of mental health problems/disorders at work and related outcomes	Covered in principle but not effectively addressed	Only implicit acknowledgement/coverage of mental health problems/disorders at work and related outcomes	Partial acknowledgement/coverage of mental health problems/disorders at work and related outcomes	Sufficient coverage but lack of specificity on mental health problems/disorders at work and related outcomes	Comprehensive coverage of mental health problems/disorders at work and related outcomes

Coverage of risk assessment aspects in relation to mental health in the workplace	No reference to or acknowledgement/coverage of risk assessment aspects in relation to mental health in the workplace	Covered in principle but not effectively addressed	Only implicit acknowledgement/coverage of risk assessment aspects in relation to mental health in the workplace	Partial acknowledgement/coverage of risk assessment aspects in relation to mental health in the workplace	Sufficient coverage but lack of specificity on risk assessment aspects in relation to mental health in the workplace	Comprehensive coverage of risk assessment aspects in relation to mental health in the workplace

Coverage of preventive actions in relation to mental health in the workplace	No reference to or acknowledgement/coverage of preventive actions in relation to mental health in the workplace	Covered in principle but not effectively addressed	Only implicit acknowledgement/coverage of preventive actions in relation to mental health in the workplace	Partial acknowledgement/coverage of preventive actions in relation to mental health in the workplace	Sufficient coverage but lack of specificity on preventive actions in relation to mental health in the workplace	Comprehensive coverage of preventive actions in relation to mental health in the workplace

**Table 2:** Policy scorecard: regulatory instruments of relevance to mental health and psychosocial risks in the workplace at the European level.

Instrument	Mental health in the workplace referred to in the objectives/scope of the policy	Coverage of exposure factors in relation to mental health in the workplace	Coverage of mental health problems/disorders at work and related outcomes	Coverage of risk assessment aspects in relation to mental health in the workplace	Coverage of preventive actions in relation to mental health in the workplace	Overall (max. 25)
(1)** Directive 89/391/EEC **the European Framework Directive on Safety and Health at Work	2	3	0	4	4	**13**

(2)** Directive 2010/32/EU** implementing the framework agreement on prevention from sharp injuries in the hospital and healthcare sector concluded by HOSPEEM and EPSU	0	5	1	5	2	**13**

(3)** Directive 2003/88/EC **concerning certain aspects of the organisation of working time (consolidates and repeals Directive 93/104/EC)	1	3	2	3	3	**12**

(4)** Directive 90/270/EEC **the minimum safety and health requirements for work with display screen equipment (fifth individual Directive within the meaning of Article 16 (1) of Directive 89/391/EEC)	3	3	0	3	2	**11**

(5)** Directive 92/85/EC** on pregnant workers and women who have recently given birth or are breast-feeding	3	3	0	3	1	**10**

(6)** Directive 94/33/EC** on the protection of young people at work	3	2	0	2	1	**8**

(7)** C155 **Occupational Safety and Health Convention (ILO), 1981	3	2	0	1	1	**7**

(8)** Directive2000/78/EC** establishing a general framework for equal treatment in employment and occupation	0	2	0	2	3	**7**

(9)** C 183** Maternity Protection Convention (ILO), 2000	0	2	0	2	3	**7**

(10)** C 181 **Private Employment Agencies Convention (ILO), 1997	0	4	0	2	1	**7**

(11)** Directive 2006/54/EC** on the implementation of the principle of equal opportunities and equal treatment of men and women in matters of employment and occupation	0	2	0	2	3	**7**

(12)** Directive 2002/14/EC** establishing a general framework for informing and consulting employees in the European Community	0	1	0	2	2	**5**

(13)** Directive 2002/15/EC** on the organisation of working time of persons performing mobile road transport activities	0	1	1	1	2	**5**

(14)** C187 **Promotional Framework for Occupational Safety and Health Convention (ILO), 2006	0	1	1	1	2	**5**

(15)** Directive 96/34/EC **on the framework agreement on parental leave	0	1	0	0	3	**4**

(16)** Directive 2000/43/EC **implementing the principle of equal treatment between persons irrespective of racial or ethnic origin	0	1	0	1	2	**4**

(17)** Directive 2009/104/EC **concerning the minimum safety and health requirements for the use of work equipment by workers at work (second individual Directive within the meaning of Article 16 (1) of Directive 89/391/EEC) [*replacing Directive 89/655/EEC*]	0	1	1	1	1	**4**

(18)** Directive 2008/94/EC** on the protection of employees in the event of the insolvency of their employer (repealing Directive 2002/74/EC and Council Directive 80/987/EEC)	0	1	0	1	1	**3**

(19)** Directive 98/59/EC **on the approximation of the laws of the member states relating to collective redundancies	0	1	0	1	1	**3**

(20)** Directive 92/91/EEC** concerning the minimum requirements for improving the safety and health protection of workers in the mineral-extracting industries through drilling (eleventh individual Directive within the meaning of Article 16 (1) of Directive 89/391/EEC)	0	1	0	1	1	**3**

(21)** Directive 92/104/EEC** on the minimum requirements for improving the safety and health protection of workers in surface and underground mineral-extracting industries (twelfth individual Directive within the meaning of Article 16 (1) of Directive 89/391/EEC)	0	1	0	1	1	**3**

(22) ** Directive 89/654/EEC** concerning the minimum safety and health requirements for the workplace (first individual directive within the meaning of Article 16 (1) of Directive 89/391/EEC)	0	1	0	1	0	**2**

(23)** Directive 89/656/EEC **on the minimum health and safety requirements for the use by workers of personal protective equipment at the workplace (third individual directive within the meaning of Article 16 (1) of Directive 89/391/EEC)	0	1	0	1	0	**2**

(24)** Directive 90/269/EEC **on the minimum health and safety requirements for the manual handling of loads where there is a risk particularly of back injury to workers (fourth individual Directive within the meaning of Article 16 (1) of Directive 89/391/EEC)	0	1	0	1	0	**2**

(25)** C175 **Part-time Work Convention (ILO), 1994	0	1	0	1	0	**2**

(26)** Directive 97/81/EC** concerning the framework agreement on part-time work	0	1	0	0	1	**2**

(27)** Directive 99/70/EC** concerning the framework agreement on fixed-term work	0	1	0	1	0	**2**

(28)** Directive 2000/79/EC** concerning the European Agreement on the Organisation of Working Time of Mobile Workers in Civil Aviation	0	1	0	0	1	**2**

(29) **Council Directive 2001/23/EC **on the approximation of the laws of the member states relating to the safeguarding of employees' rights in the event of transfers of undertakings, businesses or parts of undertakings or businesses	0	1	0	0	1	**2**

(30)** Directive 2002/73/EC **on equal treatment for men and women as regards access to employment, vocational training and promotion, and working conditions (amending Directive 76/207/EEC)	0	1	0	0	1	**2**

(31)** Directive 2009/38/EC** on the establishment of a European Works Council or a procedure in Community-scale undertakings and Community-scale groups of undertakings for the purposes of informing and consulting employees (recast)	0	1	0	0	1	**2**

(32)** Directive 93/103/EC** concerning the minimum safety and health requirements for work on board fishing vessels (thirteenth individual Directive within the meaning of Article 16 (1) of Directive 89/391/EEC)	0	1	0	0	1	**2**

(33) **Directive 92/57/EEC** on the implementation of minimum safety and health requirements at temporary or mobile construction sites (eighth individual Directive within the meaning of Article 16 (1) of Directive 89/391/EEC)	0	1	0	0	1	**2**

(34)** Directive 91/383/EEC** supplementing the measures to encourage improvements in the safety and health at work of workers with a fixed-duration employment relationship or a temporary employment relationship	0	1	0	1	0	**2**

**Table 3:** Policy scorecard: voluntary policy initiatives of relevance to mental health and psychosocial risks in the workplace.

Document	Mental health in the workplace referred to in the objectives and scope of the policy	Coverage of exposure factors in relation to mental health in the workplace	Coverage of mental health problems at work and related outcomes	Coverage of risk assessment aspects in relation to mental health in the workplace	Coverage of preventive actions in relation to mental health in the workplace	Overall (max. 25)
(1)** Guidance: EC, 1999 **Guidance on Work-Related Stress—Spice of Life or Liss of Death?	4	5	5	5	5	**24**

(2)** Guidance: EU-OSHA, 2002** How to Tackle Psychosocial Issues and Reduce Work-Related Stress	4	5	5	5	5	**24**

(3)** Guidance: WHO, 2008** PRIMA-EF: Guidance on the European Framework for Psychosocial Risk Management: A Resource for Employers and Worker Representatives	4	5	5	5	5	**24**

(4)** Guidance: ILO, 1986** Psychosocial Factors at Work: Recognition and Control	4	5	5	5	5	**24**

(5)** Guidance: ILO, 2012 **SOLVE Approach	4	5	5	5	5	**24**

(6)** Guidance: WHO, 2003,** Work Organization and Stress	4	5	5	4	5	**23**

(7)** WHO Healthy Workplaces Framework, 2010,** Healthy Workplaces: A Model for Action: For Employers, Workers, Policymakers and Practitioners	4	5	4	4	5	**22**

(8)** WHO Mental health declaration for Europe**, **2005, **and Mental Health Action Plan for Europe	5	4	4	4	4	**21**

(9) **WHO European Mental Health Action Plan, 2013**	4	5	5	3	4	**21**

(10)** Guidance: ILO, 2012, **Stress Prevention at Work Checkpoints**: **Practical Improvements for Stress Prevention in the Workplace	4	5	4	4	4	**21**

(11)** EU High-level Conference, Brussels, 2010**, Investing into well-being at Work: Managing Psychosocial Risks in Times of Change	4	5	3	4	4	**20**

(12)** Committee of Senior Labour Inspectors (SLIC), 2012,** Campaign on Psychosocial Risks at Work	4	4	3	4	3	**18**

(13)** Communication from the Commission COM (2014) 332 **on an EU Strategic Framework on Health and Safety at Work 2014–2020	4	4	3	3	4	**18**

(14)** Framework Agreement on Work-related Stress**, **2004** European Social Partners—ETUC, UNICE (BUSINESSEUROPE), UEAPME, and CEEP	3	4	3	3	4	**17**

(15)** Communication from the Commission **{**SEC (2007) 214–216**} Improving quality and Productivity at Work: Community Strategy 2007–2012 on Health and Safety at Work	4	3	3	3	4	**17**

(16)** EU-Conference, Berlin, 2011**-Promoting Mental Health and Well-Being in Workplaces	4	4	3	3	3	**17**

(17**) EN ISO 10075-1: 1991 **Ergonomic principles Related to Work-Load–General Terms and Definitions	2	4	3	3	4	**16**

(18)** R194 revised annex, ILO 2010,** Recommendation concerning the List of Occupational Diseases and the Recording and Notification of Occupational Accidents and Diseases	4	4	4	3	N/A	**15**

(19)** EN ISO 10075-2: 1996** Ergonomic Principles Related to Work-Load–Design Principles	2	3	3	3	4	**15**

(20)** Opinion of the European Economic and Social Committee, 2013, **on the European Year of Mental Health—Better Work, Better Quality of Life (2013/C 44/06)	4	4	1	3	3	**15**

(21)** Council of the European Union Conclusions, 2002,** on combating stress and depression-related problems	3	2	4	1	4	**14**

(22)** European Pact for Mental Health and Well-being, 2008,** Together for Mental Health and Well-being	3	3	2	3	3	**14**

(23)** European Parliament resolution T6-0063/2009** on Mental Health, Reference 2008/2209 (INI), Nonlegislative Resolution	3	3	2	3	3	**14**

(24)** Green paper – EC, 2005, **Improving the Mental Health of the Population: Towards a Strategy on Mental Health for the European Union	3	2	3	2	3	**13**

(25)** European Parliament Resolution 2006/2058 (INI) **on Improving the Mental Health of the Population: Towards a Strategy on Mental Health for the European Union	3	3	2	2	3	**13**

(26)** Guidance: WHO, 2007** Raising Awareness of Stress at Work in Developing Countries: a Modern Hazard in a Traditional Working Environment: Advice to Employers and Worker Representatives	3	3	2	2	2	**12**

(27)** Guidance: EU-OSHA, 2011 **Workplace Violence and Harassment: a European Picture	2	2	2	3	3	**12**

(28)** Guidance: WHO, 2003** Raising Awareness to Psychological Harassment at Work	2	2	2	2	3	**11**

(29)** Charter of Fundamental Rights of the European Union (2000/C 364/01)**	2	4	1	0	4	**11**

(30)** Guidance: ILO, 2006** Violence at Work	2	2	2	2	3	**11**

(31)** Communication from the Commission COM (2010) 682 **An Agenda for New Skills and Jobs: A European Contribution towards Full Employment	4	3	1	2	1	**11**

(32)** WHO Action Plan, 2012 **for Implementation of the European Strategy for the Prevention and Control of Noncommunicable Diseases 2012–2016	1	3	1	3	3	**11**

(33)** Council of the European Union Conclusions, 2003 **on Mental health–Conference on Mental Illness and Stigma in Europe: Facing Up the Challenges of Social Inclusion and Equity	3	2	2	1	2	**10**

(34)** Council of the European Union Conclusions, 2005 **on a Community Mental Health Action–Outcome of Proceedings	3	2	1	1	3	**10**

(35)** Opinion of the European Economic and Social Committee, 2005 **on the Green Paper Improving the Mental Health of the Population—Towards a Strategy on Mental Health for the European Union (2006/C 195/11)	3	1	1	1	2	**8**

(36)** EC 2007-White paper- **Together for Health—A Strategic Approach for the EU 2008–2013	2	1	1	3	1	**8**

(37)** Framework Agreement on Harassment and Violence at Work**, **2007, ** European Social Partners-ETUC, BUSINESSEUROPE, UEAPME, and CEEP	1	3	1	1	2	**8**

(38)** The Standing Committee of European Doctors (CPME) Position Paper, 2009, **Mental Health in Workplace Settings “Fit and Healthy at Work”	3	2	1	0	2	**8**

(39)** Council Resolution 2000/C86/01 **on The Promotion of Mental Health	3	0	2	0	2	**7**

(40)** Mental and Physical Health Platform (MPHP) 2009, **The Mental and Physical Health Charter and Call for Action	3	1	1	0	1	**6**

(41)** Recommendations from Mental Health Europe (MHE), 2009 **Work Programme of the Spanish-Belgian-Hungarian Trio Presidency of the Council of the EU (2010-2011)	3	1	1	0	1	**6**

(42)** Recommendations of the European Parliament and of the Council, 2006, **on key competences for lifelong learning	1	2	1	0	1	**5**

(43)** Council of the European Union Conclusions, 2011,** on “The European Pact for Mental Health and Wellbeing-Results and Future Action”	2	0	0	1	2	**5**

(44)** Council Decision 2003/C 218/01, ** on Setting Up an Advisory Committee on Safety and Health at Work	1	1	0	1	1	**4**

(45)** Council Resolution 2000/C218/03, **on action on health determinants	1	1	0	0	1	**3**

(46)** Council of the European Union, 2000, **Lisbon Strategy: to become the most competitive and dynamic knowledge-based economy in the world capable of sustainable economic growth with more and better jobs and greater social cohesion	0	1	0	1	1	**3**

(47)** Council of the European Union Conclusions, 2001,** on a Community strategy to reduce alcohol-related harm	1	1	0	0	1	**3**

(48)** Framework Agreement on Telework, 2002, **European social partners—ETUC, UNICE (BUSINESSEUROPE), UEAPME, and CEEP	0	1	0	1	1	**3**

(49)** Opinion of the Committee of the Regions, 2006, **on the Proposal for a Recommendation of the European Parliament and of the Council on Key Competences for Lifelong Learning	0	2	0	0	1	**3**

(50)** Commission Recommendation 2008/867/EC **on the active inclusion of people excluded from the labour market	0	1	1	0	1	**3**

(51)** Guidance: European Commission, 2009, **Report of Ad Hoc Expert Group on the Transition from Institutional to Community-based Care	1	1	0	0	1	**3**

(52)** Framework Agreement on Inclusive Labour Markets, 2010 **European social partners—ETUC, UNICE (BUSINESSEUROPE), UEAPME, and CEEP	0	2	0	0	1	**3**

(53)** Communication from the Commission COM (2010) 2020 **EUROPE 2020: A Strategy for Smart, Sustainable, and Inclusive Growth	0	2	0	0	1	**3**

(54)** Council of the European Union Conclusions, 2011,** on closing health gaps within the EU through concerted action to promote healthy lifestyle behaviours	1	1	0	0	1	**3**

(55)** Council Resolution 2000/C218/02, **on the balanced participation of women and men in family and working life	0	1	0	0	1	**2**

(56)** Framework of Actions for the Lifelong Development of Competencies and Qualifications**, **2002** European social partners—ETUC, BUSINESSEUROPE, UEAPME, and CEEP	0	1	0	0	1	**2**

(57)** Framework of Actions on Gender Equality, 2005, **European social partners—ETUC, UNICE (BUSINESSEUROPE), UEAPME, and CEEP	0	1	0	0	1	**2**

(58)** EC 2007 - White paper **on a Strategy for Europe on Nutrition, Overweight and Obesity Related Health Issues	0	1	0	0	1	**2**

(59)** Opinion of the Committee of the Regions 2008 **on Flexicurity	0	1	0	0	1	**2**

(60)** WHO European Mental Health Strategy**, **2011**	1	0	0	1	0	**2**
